# Sciatic Nerve Palsy following Total Hip Replacement: Are Patients Personal Characteristics More Important than Limb Lengthening? A Systematic Review

**DOI:** 10.1155/2017/8361071

**Published:** 2017-11-15

**Authors:** Marcello De Fine, Matteo Romagnoli, Stefano Zaffagnini, Giovanni Pignatti

**Affiliations:** ^1^General Orthopaedic Surgery, Rizzoli Sicilia Department, Rizzoli Orthopaedic Institute, Bagheria, Palermo, Italy; ^2^Clinica Ortopedica e Traumatologica II, Rizzoli Orthopaedic Institute, Bologna, Italy

## Abstract

Sciatic nerve palsies are rare but potentially devastating complications, accounting for more than 90% of neurologic injuries following total hip replacement. A systematic literature screening was carried out searching papers evaluating an exclusive population of postarthroplasty sciatic nerve palsies to ascertain (1) the influence of limb lengthening itself on sciatic nerve palsy, (2) the most important risk factors, (3) the long-term prognosis, and (4) the outcomes of different treatments. Fourteen manuscripts were finally included. The wide prevalence of retrospective case series decreased the global methodological quality of the retrieved papers. A hazardous lengthening threshold cannot be surely identified. Developmental dysplasia of the hip and previous hip surgeries are the most frequently recognized risk factors. Rate of full nerve function restoration approximates two-thirds of the cases, independently of the extent of initial neural damage. Poor evidences are available about the best treatment strategy. Well-structured multicentric prospective comparative studies are needed to substantiate or contrast the finding of this review. Anyway, since the onset of palsies is probably due to a combination of individual factors, risk of nerve damage and potential for nerve recovery should be evaluated on an individual basis.

## 1. Introduction

Neurologic injuries following total hip replacement (THR) are rare but potentially devastating complications, since neurologic pain and a variable extent of muscle weakness can frustrate an otherwise excellent clinical result, causing patients dissatisfaction and surgeons distress. Sciatic nerve palsies account for more than 90% of neurologic injuries following THR [1]. Although the occurrence of sciatic nerve palsies is uncommon [2–4], the projected increase in demand for THR [5] and the reported higher incidence of such injuries following revision surgery [6–8] further enhance the relevance of this problem. The etiology of iatrogenic sciatic nerve injuries is frequently unknown, and direct surgical lesions of the nerve are rarely involved. Traction upon the nerve, compression from subfascial hematoma, and thermal burns from extraneous cement represent the most commonly reported causes [1]. The existing literature about this fundamental topic is contrasting and confusing, and many concerns remain about risk factors, treatment, and prognosis of postarthroplasty sciatic nerve injuries. Despite excessive limb lengthening has been historically emphasized as the key factor in the onset of nerve disease, a clear correlation between limb lengthening and nerve injury is lacking, so that the influence of limb lengthening itself and the amount of dangerous lengthening are questionable [9]. Besides, conservative management and surgical exploration have been alternatively advocated as the best treatment option, and the timing of surgical operation is still under debate. The most relevant risk factors should be clearly stated, and finally, data about the long-term prognosis of these lesions are contrasting. The available literature was therefore screened in a systematic fashion aiming to ascertain (1) the influence of limb lengthening itself on sciatic nerve palsies following THR, (2) the most important reported risk factors, (3) the long-term prognosis, and (4) the outcomes of different treatment strategies in order to improve therapeutic protocols and outcomes after an acute sciatic nerve injury.

## 2. Materials and Methods

A systematic search was conducted according to the PRISMA guidelines (http://www.prisma-statement.org/). The keywords “total hip replacement/hip prosthesis” were matched with “sciatic nerve palsy”, “common peroneal nerve palsy”, “motor nerve palsy”, “sciatic nerve injury”, and “nerve palsy”, taking into account only papers in English. Grey literature was not included. No limits regarding the publication date were supplied. PubMed (https://www.ncbi.nlm.nih.gov/sites/entrez/), Ovid (http://www.ovid.com/), and Google Scholar were all queried for articles dealing with sciatic nerve palsies following THR and specifically focusing on the effect of limb lengthening, treatment options, risk factors, and long-term prognosis.

The inclusion criteria were therefore as follows: papers evaluating an exclusive population of postarthroplasty sciatic nerve palsies and dealing with the influence of limb lengthening on the onset of palsy, or with treatment strategies, risk factors, and long-term prognosis.

Case reports, instructional courses, and literature reviews were excluded as well as biomechanical analysis, cadaver studies, surgical techniques, and letters to the editors. Two authors independently evaluated the retrieved studies, judging their relevance to the topic at hand on the basis of the abstract. The title or the full text version was alternatively used when the abstract was missing. Any doubt about inclusion was resolved by the senior author.

**Figure 1:**
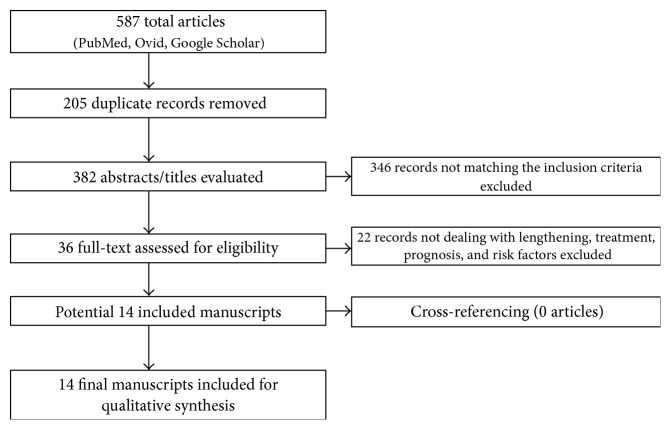
Search strategy.

From a total number of 587 retrieved articles, after duplicates elimination and abstract/title evaluation, 551 articles failed to meet the inclusion criteria. The full text of the remaining 36 articles was obtained, and cross-referencing these manuscripts, no further articles regarding the subject of the research were included. The contents of these 36 records were then screened finding 22 studies not dealing with the correlation between limb lengthening and sciatic nerve palsy, or with treatment, prognosis, and risk factors. Given the search strategy, 14 manuscripts were finally available for the review [2–4, 6, 7, 10–18] (Figure 1). The data from these studies were reported onto an anonymous data form by one of us. Each study was reviewed in detail by two of us, and study design, level of evidence, demographics, surgical approaches, years of operations, rates and types of previous hip surgeries, follow-up, and type and extent of neurological lesions were all reported on. Since all but one [16] of the studies had observational design, GRACE checklist was used to score methodology [19–21]. GRACE checklist is a validated series of 11 questions useful to assess the quality of observational studies. Two authors independently rated the manuscripts by using this tool, and any disagreement was resolved by consensus.

## 3. Results

The 14 retained articles have been published throughout a very long period. Most of these papers are retrospective case series, principally dealing with the relationship between sciatic nerve injuries and limb lengthening or with the prognosis of such injuries. The sample size was always small, ranging from 6 to 56 cases (Table 1). There were two level II studies [15, 16], and 12 level IV studies [2–4, 6, 7, 10–14, 17, 18]. A total number of 385 sciatic nerve injuries were reported on. Obviously, a variety of different surgical approaches and implant models have been used, with different preoperative diagnosis leading to THRs. Methodological scoring revealed the global poor quality of the retrieved articles, due to the large prevalence of retrospective case series and the retrospective design of the only two case-control studies [15, 16] (Table 1).

### 3.1. Lengthening

The influence of limb lengthening on the onset of sciatic nerve injuries was investigated in seven articles [2–4, 7, 11, 13, 16], but the average lengthening was recorded just in four [3, 4, 13, 16]. In the paper by Schmalzried et al. [7], lengthening was assessed considering both sciatic and femoral nerve palsies, thus making data exclusively pertaining to sciatic nerve injuries indistinguishable (Table 2). Average lengthening was reported just in four papers [3, 4, 13, 16], ranging from 0,3 to 1,9 cm. The rate of previous hip surgery ranged from 12% to 43% of the cases. In four papers the lengthened hips were categorized using a 2 cm threshold [2, 3, 11, 13]. In these four papers, the rate of lengthened hips and the rate of previous hip surgery seem to be somewhat similar (Table 2).

### 3.2. Risk Factors

A statistical evaluation of significant risk factors was present in seven papers [6, 7, 10, 12, 13, 15, 16] (Table 3). Developmental dysplasia of the hip and previous hip surgery are the most frequently recognized risk factors, whereas limb lengthening was found to be related to nerve injury just in one paper.

### 3.3. Prognosis

As it could be expected, the potential for nerve recovery was the most frequently investigated issue [3, 4, 6, 7, 10–13, 15, 16, 18], notwithstanding the necessary follow-up (minimum two years or until complete neurological recovery), and the exact evaluation of the rate of complete lesions was present just in four articles [13, 15, 16, 18] (Table 4). In these four papers the rate of recovery approximates two-thirds of the cases. Due to its anatomical location, common peroneal nerve injuries were most often involved, reaching a cumulative rate of 66% among the 8 articles investigating this topic [3, 4, 6, 7, 12, 13, 15, 16].

### 3.4. Treatment

Three papers evaluated the outcomes of surgically treated sciatic nerve injuries [13, 14, 17], reporting on a total of 15 cases. The poor number of patients enrolled does not permit any conclusion about this issue.

## 4. Discussion

The present systematic review tried to summarize the available literature about postarthroplasty sciatic nerve injuries with the aim of ascertaining (1) the influence of limb lengthening itself on sciatic nerve palsies following THR, (2) the most important reported risk factors (3), the long-term prognosis, and (4) the outcomes of different treatment strategies in order to improve therapeutic protocols and outcomes after an acute sciatic nerve injury.

We need to point out the several and hard limitations of this work.

First of all, we must highlight the poor quality of the retrieved articles, mostly involving level IV studies, with low global methodological scoring (Table 1). Besides, small sample sizes were almost always taken into account, and a total number of 385 sciatic injuries have been assessed. The wide variability and the substantial heterogeneity of the extracted data precluded the pooled analysis of the results. In fact, just a qualitative synthesis was carried out. These drawbacks must be ascribed to the rarity of sciatic nerve injuries, and only well-structured long-lasting multicentric studies could face the problem in a prospective comparative fashion, ensuring an adequate population. As a matter of fact, such a powerful type of study is lacking among the current literature; nevertheless, hip arthroplasty malpractice claims are constantly growing. Sciatic nerve injury is one of the main sources for litigation after THA in the United States [22, 23], accounting for 19.6% of all orthopaedics claims in the Netherlands [24]. Obviously, the large prevalence of case series and the methodological flaws of the retained articles impair the significance of this review; anyway, we judge that a systematic literature search is essential at present to outline the state of the art about postarthroplasty sciatic nerve palsies in view of the central role of this complication in the orthopaedics malpractice claims worldwide [25].

The results of this study showed that lengthening itself seems not to play a central role in nerve dysfunction, and DDH and previous hip surgery are the most relevant risk factors for the onset of sciatic injuries. Besides, the extent of neural damage seems not to correlate with the likelihood of complete recovery, since full nerve function restoration was recorded in one-third to two-thirds of the cases, independently of the rate of complete lesions. Finally, no evidences have emerged about the best treatment strategy.

Although rarely occurring, sciatic nerve injuries can significantly impair the clinical success of an otherwise excellent THR procedure. Besides, the steady increase in THR utilization worldwide and the subsequent increase in revision hip surgery [26] overstate the relevance of this issue.

Sciatic nerve palsies are the most common neurologic injuries following THA, with reported incidences ranging from 0.6 to 3.8% [1]. The precise etiology of nerve damage is unknown in about 50% of the cases [2, 7], and limb lengthening has been traditionally considered the most relevant causative factor [1, 3] due to the well-known limited nerves resistance to stretches [27, 28]. The presence of a dense scar tissue embedding the nerve, as in cases of previously operated hips, should probably result in a further reduction of nerve's elasticity, facilitating the occurrence of nerve injuries in reoperated hips.

### 4.1. Lengthening

Previous historical reports and empirical evidences suggest that three- to four-centimeter lengthening poses the higher risk of neurologic injuries [3]; however, even in the past doubts about the role of lengthening were present. Nercessian et al. [29] evaluating 1284 hip prostheses, except for one iatrogenic lesion, found no sciatic nerve injuries even in extremely lengthened hips. As it was clearly visible in Table 2, when available [3, 4, 13, 16], the reported average lengthening seems not to be so excessive, ranging from 0,3 to 1,9 cm, and in 2 out of these four articles [4, 16] even range of lengthening does not exceed 2,5 cm. When a 2 cm threshold was applied [2, 3, 11, 13], just in one old paper the rate of lengthened hips appears to be relevant [3]. Interestingly, in these four articles the rate of lengthened hips approximates the rate of reoperated hips.

### 4.2. Risk Factors

The most relevant risk factors for postarthroplasty sciatic nerve injuries were reported in Table 3.

Preoperative diagnosis of developmental dysplasia of the hip (DDH) or previous hip surgery was the most frequently recognized significant risk factor [7, 12, 13, 15]. Lengthening was considered a significant risk factor just in one paper [15], but this is one of the only two case-control studies included in this review.

### 4.3. Prognosis

Long-term prognosis of sciatic nerve injuries was the most frequently investigated issue (Table 4) but only four papers reported on the adequate minimum follow-up and the percentage of complete lesions [13, 15, 16, 18]. The more lateral position, the tethering at both the sciatic notch and the peroneal head, and the thinner connective tissue coverage in respect to the tibial division should make the peroneal nerve more susceptible to iatrogenic injuries [27, 28]; nonetheless, the prevalence of peroneal division injuries was evident just in two out of these four papers [15, 16]. Since the work by Schmalzried et al. [7], the extent of neural injury was thought to correlate with the possibility of full nerve function restoration. Despite this, the percentage of full recovery ranges from one-third to two-thirds of the cases, independently of the rate of complete lesions [13, 15, 16, 18] (Table 4).

Zappe and coauthors [18], evaluating 9 sciatic nerve injuries, recorded 4 complete lesions, one of which (25%) fully recovered after 5 years. In the paper by Pekkarinen et al. [13] the rate of full recovery for complete peroneal division lesions was 33% (7 out of 21 lesions) and 20% (2 out of 10 lesions) for complete tibial division lesions, but no mentions were found about the timing of recovery.

Park et al. [16] reported a 60% (3 out of five) rate of full recovery in complete lesions at a mean of 14.5 months (range, 8 to 21 months). The authors [16] claimed that body mass index rather than the extent of motor nerve involvement had a significant correlation with the chance of full recovery. A 38% rate of full recovery at an average 21 months follow-up (10 out of the 26 patients available for follow-up) was registered by Farrell et al. [15]. Interestingly, a similar rate of recovery was found for incomplete lesions.

### 4.4. Treatment

Our efforts toward the detection of an effective treatment algorithm were unsuccessful. The only three papers evaluating the outcomes of surgically treated sciatic nerve injuries reported on just 15 cases and on this basis any conclusion would be anecdotal. In our opinion the flow chart proposed by Kyriacou et al. [17], claiming surgical exploration of the nerve in cases of neuropathic pain or documented hematoma, could represent an interesting suggestion, but data are needed to substantiate this approach.

Even accepting the noticeable limitations of the present work, such as the general poor quality of the retrieved articles, the lack of prospective comparative studies, the small number of cases evaluated, and the lack of pooled analysis of the results, limb lengthening itself seems not to play a central role in nerve dysfunction. Since the real aetiology of nerve damage is unknown in the majority of the cases, and considering the results of this systematic review, failing to highlight a hazardous lengthening threshold, evidences about the causative role of limb lengthening are lacking to date. In other words, if sciatic nerve palsy occurs after THA with a lengthened limb, how can one demonstrate that palsy is due to lengthening rather than hematoma, traction maneuvers, retractor placement, and so on?

DDH and previous hip surgeries represented the most frequently reported risk factors. Limb lengthening often occurs when THR is planned on dysplastic hips, due to disturbed hip anatomy with generally shortened legs [30–33]. Even in cases of revision hip surgery, limb lengthening is often required to compensate for previous shortening. Retracting scar tissue from previous operations, or the altered anatomic location of the nerve in DDH, can probably reduce nerve's elasticity, with an increased probability of iatrogenic damage. The available literature does not permit identifying a lengthening threshold.

Besides, this systematic literature review was undertaken to detail prognosis of postarthroplasty sciatic nerve injuries. In contrast with the findings of Schmalzried and colleagues [7], the extent of neural damage seems not to correlate with the likelihood of complete recovery. Full nerve function restoration was recorded in one-third to two-thirds of the cases, independently of the rate of complete lesions [13, 15, 16, 18]. Our last goal was to identify the best treatment strategy, but unfortunately no evidences are available on this topic.

In our opinion, the onset of postarthroplasty sciatic nerve injuries cannot be generally ascribed to a single cause but is probably related to a combination of individual factors. Nerve's stretching due to lengthening is likely to result in a palsy if an altered proximal femoral anatomy is concomitant, such as in cases of DDH, or in the presence of retracting scar tissue, as in reoperated hips. Even the prognosis of nerve lesions appears to be widely different and independent from the extent of initial damage, being seemingly correlated to patients' specific features, as body mass index. Finally, in the absence of guidelines, treatment of nerve lesions should be tailored to each patient.

## 5. Conclusion

On the basis of the scrutinized articles, we can affirm that DDH and previous hip surgery are the most relevant risk factors for postarthroplasty sciatic nerve injuries, whereas a hazardous lengthening threshold cannot be surely identified. Patients should be informed on the poor long-term prognosis of such lesions, since full nerve recovery can be expected in one-third to two-thirds of the cases, generally after a long time. Well-structured multicentric prospective comparative studies are needed to substantiate or contrast the finding of this review. Anyway, risk of nerve damage and potential for nerve recovery should be evaluated on an individual basis.

## Figures and Tables

**Table 1:** Available literature about postarthroplasty sciatic nerve injuries.

Authors, year	Level of Evidence	GRACE score (number of items)	Topics assessed	Number of sciatic injuries
Weber et al., 1976	IV	2/11	Prognosis, risk factors	10
Johanson et al., 1982	IV	3/11	Lengthening	34
Edwards et al., 1987	IV	3/11	Lengthening, prognosis	23
Schmalzried et al., 1991	IV	5/11	Lengthening, prognosis, risk factors	48
Simon et al., 1993	IV	4/11	Lengthening, prognosis	16
Nercessian et al., 1994	IV	8/11	Lengthening, prognosis	29
Navarro et al., 1995	IV	5/11	Prognosis, risk factors	7
Oldenburg et al., 1997	IV	4/11	Prognosis, risk factors	46
Pekkarinen et al., 1999	IV	9/11	Lengthening, prognosis, treatment, risk factors	27
Butt et al., 2005	IV	4/11	Treatment	6
Farrell et al., 2005	II	9/11	Prognosis, risk factors	44
Park et al., 2013	II	11/11	Lengthening, prognosis, risk factors	30
Kyriacou et al., 2013	IV	5/11	Treatment	56
Zappe et al., 2014	IV	8/11	Prognosis	9

**Table 2:** Postarthroplasty sciatic nerve injuries and limb lengthening.

Authors	Number of sciatic injuries	Average lengthening (cm)	Rate of lengthened hips	Rate of previous hip surgery
Johanson	34	N.A.	5 limbs ≥ 2 cm (15%)	7 cases (21%) (2 Girdlestone, 2 arthrodesis, 1 Colonna arthroplasty, 1 hemiarthroplasty, 1 ORIF)
Edwards	23	1,9 (range −1,5–5,1)	12 limbs ≥ 2 cm (52%)	10 cases (43%) (8 THRs, 2 arthrodesis)
Schmalzried	48	N.A.	N.C.	N.C.
Simon et al.	16	N.A.	3 limbs ≥ 2 cm (19%)	2 cases (12%) (THRs)
Nercessian	29	0,6 (range 0–2)	N.A.	9 cases (31%) (THRs)
Pekkarinen	27	1,4 (range 1–4,1)	8 limbs ≥ 2 cm (30%)	6 cases (22%) (THRs)
Park	30	0,3 (range 0–2,5)	N.A.	4 cases (13%) (THRs)

N.A.: not available; N.C.: not clear.

**Table 3:** Statistically significant recorded risk factors.

Authors	Age	Gender	DDH	Previous hip surgery	Lengthening	Others
Weber		**+**				
Schmalzried			+	+		
Navarro						
Oldenburg				+		
Pekkarinen			+	+		Fibrotic ankylosis after joint sepsis
Farrell			+		+	Posterior approach, cementless stem fixation
Park	+					

**Table 4:** Prognosis of sciatic nerve injuries.

Authors	Number of sciatic injuries	Follow-up	% complete lesions	Type of lesion	% full recovery
Weber	10	1 Year	100%	N.A.	40%
Edwards	23	Mean 2.7 years	N.A.	12 peroneal, 11 sciatic	13%
Schmalzried	48	12–198 ms	73%	26 peroneal, 19 sciatic, 3 tibial	N.C.
Simon et al.	16	N.C.	0%	N.A.	75%
Nercessian	29	Minimum 2 years	N.A.	23 peroneal, 6 sciatic	66%
Navarro	7	1–2,5 years	N.A.	6 peroneal, 1 sciatic	14%
Oldenburg	46	Mean 107 months (11 to 240)	N.A.	33 peroneal, 13 sciatic	N.C.
Pekkarinen	27	Mean 58 months (24 to 110)	78%	11 peroneal, 15 sciatic, 1 tibial	63%
Farrell	44	Mean 6 years (0.2–21 ys)^*∗*^	61%	30 peroneal, 14 sciatic	39%
Park	30	Mean 44,3 ms (3.7–114.4 ms)^*∗*^	17%	26 peroneal, 4 sciatic	57%
Zappe	9	Mean 93 ms^*∗*^	44%	N.A.	67%

^*∗*^Neurological deficit was followed until complete recovery or at least 2 years; N.A.: not available; N.C.: not clear.

## References

[1] Su E. P. (2017). Post-operative neuropathy after total hip arthroplasty. *Bone & Joint Journal*.

[2] Johanson N. A., Pellicci P. M., Tsairis P., Salvati E. A. (1983). Nerve injury in total hip arthroplasty. *Clinical Orthopaedics and Related Research*.

[3] Edwards B. N., Tullos H. S., Noble P. C. (1987). Contributory factors and etiology of sciatic nerve palsy in total hip arthroplasty. *Clinical Orthopaedics and Related Research*.

[4] Nercessian O. A., Macaulay W., Stinchfield F. E. (1994). Peripheral neuropathies following total hip arthroplasty. *The Journal of Arthroplasty*.

[5] Kurtz S., Ong K., Lau E., Mowat F., Halpern M. (2007). Projections of primary and revision hip and knee arthroplasty in the United States from 2005 to 2030. *The Journal of Bone & Joint Surgery*.

[6] Navarro R. A., Schmalzried T. P., Amstutz H. C., Dorey F. J. (1995). Surgical approach and nerve palsy in total hip arthroplasty. *The Journal of Arthroplasty*.

[7] Schmalzried T. P., Amstutz H. C., Dorey F. J. (1991). Nerve palsy associated with total hip replacement. Risk factors and prognosis. *The Journal of Bone & Joint Surgery—American Volume*.

[8] Schmalzried T. P., Noordin S., Amstutz H. C. (1997). Update on nerve palsy associated with total hip replacement. *Clinical Orthopaedics and Related Research*.

[9] Eggli S., Hankemayer S., Müller M. E. (1999). Nerve palsy after leg lengthening in total replacement arthroplasty for developmental dysplasia of the hip. *The Journal of Bone & Joint Surgery (British Volume)*.

[10] Weber E. R., Daube J. R., Coventry M. B. (1976). Peripheral neuropathies associated with total hip arthroplasty. *The Journal of Bone & Joint Surgery*.

[11] Simon J. P., Van Delm I., Fabry G. (1993). Sciatic nerve palsy following hip surgery. *Acta Orthopædica Belgica*.

[12] Oldenburg M., Müller R. T. (1997). The frequency, prognosis and significance of nerve injuries in total hip arthroplasty. *International Orthopaedics*.

[13] Pekkarinen J., Alho A., Puusa A., Paavilainen T. (1999). Recovery of sciatic nerve injuries in association with total hip arthroplasty in 27 patients. *The Journal of Arthroplasty*.

[14] Butt A. J., McCarthy T., Kelly I. P., Glynn T., McCoy G. (2005). Sciatic nerve palsy secondary to post-operative haematoma in primary total hip replacement. *The Journal of Bone & Joint Surgery B*.

[15] Farrell C. M., Springer B. D., Haidukewych G. J., Morrey B. F. (2005). Motor nerve palsy following primary total hip arthroplasty. *The Journal of Bone & Joint Surgery*.

[16] Park J. H., Hozack B., Kim P. (2013). Common peroneal nerve palsy following total hip arthroplasty: Prognostic factors for recovery. *The Journal of Bone & Joint Surgery*.

[17] Kyriacou S., Pastides P. S., Singh V. K., Jeyaseelan L., Sinisi M., Fox M. (2013). Exploration and neurolysis for the treatment of neuropathic pain in patients with a sciatic nerve palsy after total hip replacement. *The Journal of Bone & Joint Surgery (British Volume)*.

[18] Zappe B., Glauser P. M., Majewski M., Stöckli H. R., Ochsner P. E. (2014). Long-term prognosis of nerve palsy after total hip arthroplasty: results of two-year-follow-ups and long-term results after a mean time of 8 years. *Archives of Orthopaedic and Trauma Surgery*.

[19] Dreyer N. A., Schneeweiss S., McNeil B. J. (2010). GRACE principles: Recognizing high-quality observational studies of comparative effectiveness. *American Journal of Managed Care*.

[20] Dreyer N. A., Bryant A., Velentgas P. (2016). The GRACE checklist: A validated assessment tool for high quality observational studies of comparative effectiveness. *Journal of Managed Care and Specialty Pharmacy*.

[21] Dreyer N. A., Velentgas P., Westrich K., Dubois R. (2014). The GRACE checklist for rating the quality of observational studies of comparative effectiveness: a tale of hope and caution. *Journal of Managed Care Pharmacy*.

[22] Upadhyay A., York S., Macaulay W., McGrory B., Robbennolt J., Bal B. S. (2007). Medical malpractice in hip and knee arthroplasty. *The Journal of Arthroplasty*.

[23] Hofmann A. A., Skrzynski M. C. (2000). Leg-length inequality and nerve palsy in total hip arthroplasty: A lawyer awaits!. *Orthopedics*.

[24] Zengerink I., Reijman M., Mathijssen N. M. C., Eikens-Jansen M. P., Bos P. K. (2016). Hip arthroplasty malpractice claims in the Netherlands: closed claim study 2000-2012. *The Journal of Arthroplasty*.

[25] Attarian D. E., Vail T. P. (2005). Medicolegal aspects of hip and knee arthroplasty. *Clinical Orthopaedics and Related Research*.

[26] Nemes S., Gordon M., Rogmark C., Rolfson O. (2014). Projections of total hip replacement in Sweden from 2013 to 2030. *Acta Orthopaedica*.

[27] Fleming P., Lenehan B., O'Rourke S., McHugh P., Kaar K., McCabe J. P. (2003). Strain on the human sciatic nerve in vivo during movement of the hip and knee. *The Journal of Bone & Joint Surgery (British Volume)*.

[28] Borrelli J., Kantor J., Ungacta F., Ricci W. (2000). Intraneural sciatic nerve pressures relative to the position of the hip and knee: A human cadaveric study. *Journal of Orthopaedic Trauma*.

[29] Nercessian O. A., Piccoluga F., Eftekhar N. S. (1994). Postoperative sciatic and femoral nerve palsy with reference to leg lengthening and medialization/lateralization of the hip joint following total hip arthroplasty. *Clinical Orthopaedics and Related Research*.

[30] Charity J. A. F., Tsiridis E., Sheeraz A. (2011). Treatment of Crowe IV high hip dysplasia with total hip replacement using the Exeter stem and shortening derotational subtrochanteric osteotomy. *The Journal of Bone & Joint Surgery (British Volume)*.

[31] Rogers B. A., Garbedian S., Kuchinad R. A., Backstein D., Safir O., Gross A. E. (2012). Total hip arthroplasty for adult hip dysplasia. *The Journal of Bone & Joint Surgery*.

[32] Reikeras O., Haaland J. E., Lereim P. (2010). Femoral shortening in total hip arthroplasty for high developmental dysplasia of the hip. *Clinical Orthopaedics and Related Research*.

[33] Traina F., De Fine M., Biondi F., Tassinari E., Galvani A., Toni A. (2009). The influence of the centre of rotation on implant survival using a modular stem hip prosthesis. *International Orthopaedics*.

